# A cost-effectiveness analysis of ruxolitinib versus best alternative therapy for patients with steroid-refractory chronic graft-versus-host disease aged > 12 years in Singapore

**DOI:** 10.1186/s12962-023-00444-w

**Published:** 2023-05-31

**Authors:** Jian Chun Matthew Ong, Hein Than, Sandeep Tripathi, Christina Gkitzia, Xiaojun Wang

**Affiliations:** 1 Novartis Singapore Pte. Ltd., Mapletree Business City, Singapore, Singapore; 2 Department of Haematology, Singapore General Hospital, Singapore, Singapore; 3 Novartis Healthcare Pvt. Ltd., Hyderabad, India; 4 Novartis Pharma AG, Basel, Switzerland

**Keywords:** Cost, cGVHD, Allogeneic, Transplant, Ruxolitinib, QALY, Life year, Direct medical costs

## Abstract

**Background:**

Approximately 30–70% of patients who have undergone allogeneic (allo) hematopoietic stem cell transplantation (HSCT) eventually experience chronic graft-versus-host disease (cGVHD). Patients who develop steroid-refractory (SR)-cGVHD are the most severely impacted due to significant disease and financial burden. There remains an unmet need for safe, efficacious, and accessible treatments for these patients. The objective of this study was to determine the cost effectiveness of ruxolitinib for treatment of SR-cGvHD from the Singapore healthcare system perspective.

**Methods:**

Based on data from the REACH3 randomized open-label trial, a semi-Markov model was developed to evaluate cost-effectiveness of ruxolitinib compared with investigators' choice of best alternative therapy (BAT) for treatment of patients > 12 years of age with SR-cGVHD in Singapore over a 40-year time horizon. The model only considered direct medical-care costs related to the treatment of SR-cGVHD and reported them in Singapore Dollars (SGD). Half-cycle correction was applied to all costs and outcomes, which were discounted at 3%. Probabilistic sensitivity analysis (PSA), one-way sensitivity analysis (OWSA), and scenario analysis were conducted to explore the drivers of uncertainty in the model.

**Results:**

In the deterministic base case, more life years (LY; 10.28 vs. 9.42) and quality-adjusted life years (QALYs; 7.31 vs. 6.51) were gained with ruxolitinib than BAT at higher costs (SGD 303,214 vs. SGD 302,673) leading to an incremental cost-effectiveness ratio (ICER) of SGD 677/QALY. At a willingness-to-pay threshold of SGD 75,000/QALY gained, PSA found that ruxolitinib had a 78.52% probability of being cost-effective. Findings were sensitive to variations in non-responder utilities in the BAT arm and duration of BAT treatment in the OWSA, or comparison to either methotrexate (MTX) or mycophenolic acid as a single comparator in the scenario analysis. ICERs remained lower than SGD 75,000/QALY in all other tested variations and scenarios.

**Conclusion:**

Ruxolitinib is likely to be cost-effective from Singapore healthcare system’s perspective for patients with SR-cGVHD, which is promising in the management of patients with unmet clinical needs.

**Supplementary Information:**

The online version contains supplementary material available at 10.1186/s12962-023-00444-w.

## Background

Hematopoietic stem cell transplantation (HSCT) remains an important therapy for long term remission of many malignant and nonmalignant hematological disorders [1, 2]. The number of allogeneic (allo) transplantations performed annually has increased in recent years, reportedly growing by 89.0% globally and up to 193.4% in Southeast Asia/the Western Pacific region between years 2006–2016 [3]. However, allo-HSCT is a complicated and expensive procedure, particularly compounded by the challenges and costs associated with the management of its numerous associated complications.

Graft-versus-host disease (GVHD) is a complication that occurs following allo-HSCT and is a major driver of posttransplant morbidity and mortality [2]. GVHD may be classified as acute GVHD or chronic GVHD (cGVHD) based on a combination of clinical features and the time of occurrence after transplantation [4, 5]. An estimated 30–70% of allo-HSCT recipients, who survive more than 100 days after transplantation, develop cGVHD. The quality of life (QoL) of these patients is impaired, and they require continuous medical follow-ups, while facing a higher risk of infection and death [6]. Optimizing the management of cGVHD is essential to enhance treatment outcomes while minimizing psychological and financial implications for these patients [6–8].

While standard first-line treatment for cGVHD involves the use of corticosteroids, 50% of patients with cGVHD develop steroid-refractory cGVHD (SR-cGVHD) after transplantation [2, 9]. There is no consensus regarding the optimal treatment strategy for SR-cGVHD, and the choice of a standardized second-line therapy remains unclear [2, 6]. Common treatment options for SR-cGVHD include calcineurin inhibitors, extracorporeal photopheresis (ECP), ibrutinib, Janus kinase (JAK) inhibitors, mycophenolate mofetil (MMF), rituximab, mammalian target of rapamycin inhibitors, pentostatin, proteasome inhibitors, and tyrosine kinase inhibitors [2, 10, 11]. However, the effectiveness of these options varies substantially, with patients with SR-cGVHD generally facing a poor prognosis [12].

In the REACH3 (NCT03112603) trial, ruxolitinib, a potent, selective, and orally bioavailable JAK1/2 inhibitor, has shown promising efficacy in treating SR-cGVHD after allo-HSCT [9]. In this randomized open-label Phase III trial, ruxolitinib achieved higher overall response rates and duration of response (DoR) when compared against investigators’ choice of best alternative therapy (BAT). Ruxolitinib has since received approval from the Health Sciences Authority of Singapore for treating cGVHD in patients aged 12 years and older who respond inadequately to corticosteroids [13].

Cost-effectiveness is becoming a major consideration for reimbursement and healthcare resource allocation to maximise healthcare outcomes. Cost-effectiveness evidence, in addition to efficacy and safety data, influences the reimbursement decision-making process, thereby impacting the number of patients who can gain access to and benefit from novel treatments. The objective of this study is to evaluate the cost-effectiveness of ruxolitinib versus BAT from the Singapore healthcare system’s perspective.

## Methods

### Model design

**Fig. 1:**
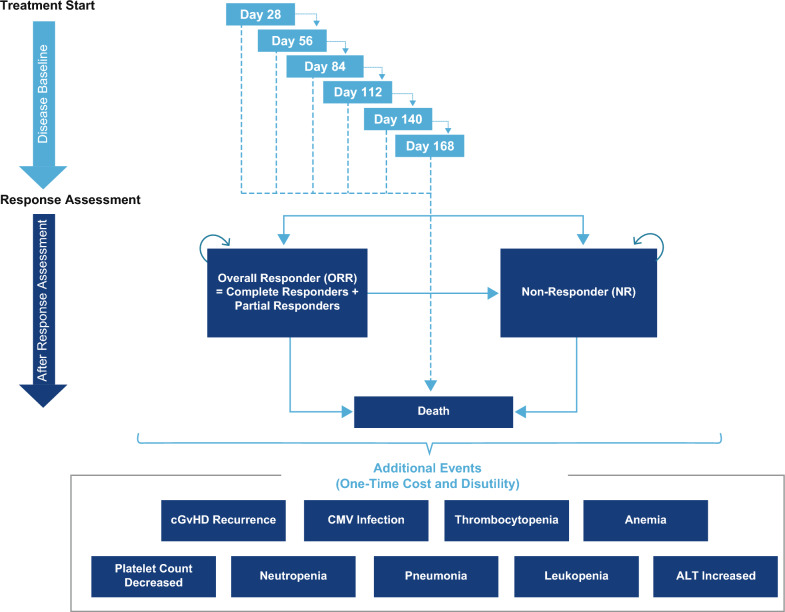
Schematic diagram of the Markov model used in this study for cost-effectiveness analysis. The arrows show the transition of patients into different health states during each model cycle. At disease baseline, patients first pass through six 28-day tunnel states to capture initial mortality. During these cycles, patients can either progress to the next tunnel state or move into the death state. At response assessment (day 168), patients were assigned into overall responder (ORR) or non-responder (NR) health states. During each subsequent 28-day cycle, patients in the ORR health state could either remain in the ORR health state, progress to the NR health state, or die. Similarly, patients in the NR health state could either remain in the same health state or die during each model cycle. ALT, alanine aminotransferase; cGVHD, chronic graft-versus-host disease; CMV, cytomegalovirus

A semi-Markov model was developed using Microsoft Excel® to capture all costs and outcomes associated with ruxolitinib and BAT for the treatment of patients with SR-cGVHD. To capture initial mortality and QoL prior to response assessment, patients first passed through a series of six 28-day tunnel states (Fig. 1). Mirroring the REACH3 trial, response to treatment was assessed on Day 168, when patients were assigned to either overall responder (ORR) or non-responder (NR) health states based on the response achieved in the respective treatment arms in the trial (Table 1).

**Table 1:** Response rate as assessed at Day 168 in the REACH3 trial

	Ruxolitinib (%)	Best alternative therapy (%)
Overall response	49.70	25.61
No response	41.21	68.29
Dead	9.09	6.10

During each subsequent 28-day cycle, patients in the ORR health state could either remain in the ORR health state, progress to the NR health state, or die. Similarly, patients in the NR health state could either remain in the same health state or die during each model cycle. Patients who progressed into the NR health state were assumed to have received BAT as subsequent treatment.

Overall survival (OS) and DoR for patients in ORR and NR health states were determined by post hoc analysis of the individual patient-level data (IPD) obtained in the REACH3 trial. Survival models (exponential, Weibull, Gompertz, log-normal, log-logistic, generalized gamma, and gamma) were fit to the IPD using R 3.6.167 and the flexsurvreg function of the flexsurv package [14]. Cox proportional hazard assumptions were tested, and pairwise hazard ratios (HRs) were also calculated and fitted. For each curve, the parameters of model fit analysis (i.e., Akaike’s information criterion [AIC] and Bayesian information criterion [BIC]) were calculated (Additional file 1: Fig. S1). The most appropriate curve for data extrapolation was selected based on the goodness-of-fit survival models with the lowest AIC and BIC. Among the curves with a good statistical fit, a clinically meaningful extrapolation of curves in the base case was ensured by excluding curves which had indefinitely extended tails (Additional file 1: Table S1).

The model was designed to capture all costs and life years (LYs) and quality-adjusted LYs (QALYs) gained. A lifetime horizon of 40 years was deemed appropriate as < 1% of patients remained alive in the parametric survival extrapolations. This 40-year time horizon is consistent with that used in other health technology assessments [15, 16].

Half-cycle correction was applied to all costs and QALYs, which were discounted at 3% on an annual basis (beginning at the end of the first year) as per recommendations by the Singapore Health Technology Assessment agency, Agency for Care Effectiveness (ACE) [17]. The model captured costs and disutilities associated with disease complications and adverse events (AEs) as one-time costs and disutilities at the median time of onset.

### Model inputs

#### Comparator choice

As some of the alternative treatment options for investigators to choose from in the BAT arm of the REACH3 trial are not routinely used in Singapore, the relevant composition of the BAT arm was determined based on local clinical practice. The composition of BAT in the current analysis included ECP (60%), rituximab (5%), MTX (15%), MMF (15%), and ibrutinib (5%).

#### Costs and resource use

Per ACE guidance [17], only direct medical-care costs related to the treatment of SR-cGVHD were considered for the analysis. The cost-effectiveness model considered drug treatment costs (based on drug acquisition costs and duration of treatment [DoT]), subsequent treatment costs, treatment administration costs, disease management costs (including hospitalizations and outpatient visits), and disease complication and AE costs in the base case (Additional file 1: Table S5). Average drug doses and DoT were based on the average weekly dosing used in the REACH3 trial and extrapolation of DoT determined therein. Costs of all the drugs and resources used were extracted from available local database, literature reviews, and publicly available cost-related resources published by ACE.

#### Health state utility

A post hoc analysis of IPD from the REACH3 trial was conducted to determine QoL associated with response to treatment. As a patient’s QoL changes over time, three sets of EuroQoL five-dimensional instrument (EQ-5D) values were considered for ORR and NR at (a) disease baseline (applies from disease baseline up to the response assessment timepoint), (b) Week 24–Week 56 (applies from Week 24 to Week 55 for each response health state), and (c) Week 56 and onward (for each response health state) (Additional file 1: Table S4).

A literature search was performed to retrieve data on the median duration and disutilities associated with each complication (Additional file 1: Table S5). The impact on QALYs was then estimated, reflecting both the utility decrement and duration of the event (Additional file 1: Table S5).

### Base-case and sensitivity analysis

#### Probabilistic sensitivity analysis

The model employed a probabilistic sensitivity analysis (PSA) to account for the joint uncertainty of the underlying parameter estimates. The common distributions used in the probabilistic analyses were beta, gamma, log-normal, normal, and Dirichlet. The choice of distribution was based on the recommendations by Briggs et al. [18]. In the absence of a formal willingness-to-pay (WTP) threshold, an implicit WTP threshold of Singapore dollar (SGD) 75,000/QALY, derived from a previous analysis of reimbursement decisions, was adopted in this study [19].

#### One-way sensitivity analysis and scenario analysis

Deterministic one-way sensitivity analysis is used to help decision makers understand the impact of changes in the value of specific parameters on model findings. A one-way sensitivity analysis (OWSA) was conducted by applying a 20% variation to the default values for all costs, utilities, proportions, and duration of AE onset, as well as parameters used for parametric survival extrapolation. Variables with the largest impact on findings were presented in a tornado diagram.

Scenario analyses were performed to test the impact of uncertainty around key model inputs and assumptions (Additional file 1: Table S6). Notable scenarios tested include alternative survival extrapolations based on treatment arm, BAT composition per the REACH3 trial, single comparisons against the three most common alternatives to Ruxolitinib as well as a societal perspective which included lost earnings from early death and lost productivity from work missed due to illness.

## Results

### Base-case

Over a 40-year time horizon, ruxolitinib was associated with incremental costs of SGD 540 compared with the BAT (SGD 303,214 vs. SGD 302,673; Table 2), 0.86 more LYs than BAT (10.28 vs. 9.42; Table 2), and 0.80 more QALYs compared with BAT (7.31 vs. 6.51; Table 2). The comparison yielded an incremental cost-effectiveness ratio (ICER) of SGD 627/LY or SGD 677/QALY. Comparison of different response outcomes is presented in Additional file 1: Fig. S1.

**Table 2:** Summary of results from the base-case analysis (discounted)

	RUX	BAT	Incremental
LYs	10.28	9.42	0.86
QALYs	7.31	6.51	0.80
Costs	SGD 303,214	SGD 302,673	SGD 540
ICER (Cost/LY)	SGD 627/LY		
ICER (Cost/QALY)	SGD 677/QALY		

BAT, best available therapy; ICER, incremental cost-effectiveness ratio; LY, life year; QALY, quality-adjusted life year; RUX, ruxolitinib; SGD, singapore dollar

### Probabilistic sensitivity analysis

**Fig. 2:**
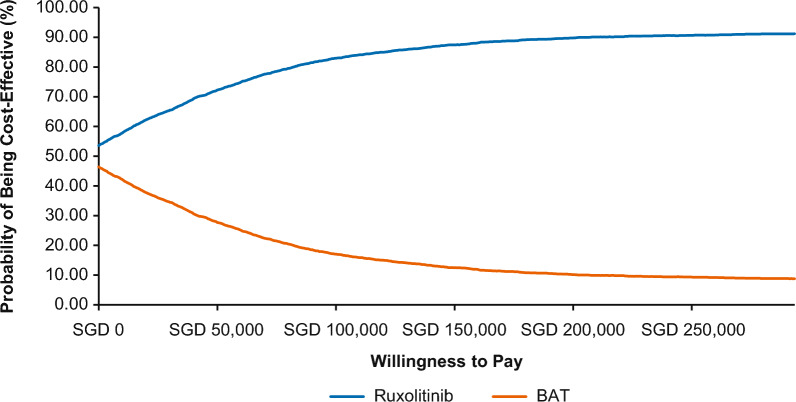
Cost-effectiveness acceptability curve. BAT, best available therapy; QALY, quality-adjusted life year; SGD, Singapore dollar

**Fig. 3:**
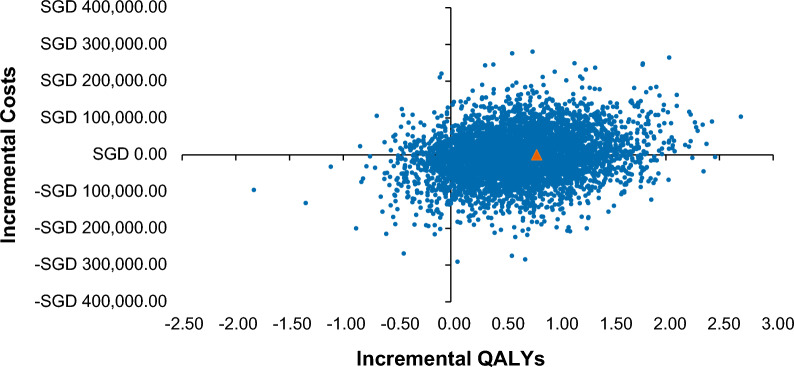
Scatter plot of probabilistic sensitivity analysis. *Note*: Orange triangle represents deterministic analysis results. QALY, quality-adjusted life year; SGD, Singapore dollar

At a WTP threshold of SGD 75,000/QALY gained, ruxolitinib had a 78.52% probability of being cost-effective compared to BAT (Fig. 2) with mean incremental costs of − SGD 4214, mean incremental LYs of 0.63, and mean incremental QALYs of 0.63. Notably, ruxolitinib dominated (more effective while costing less) BAT in 47.62% of iterations. Additionally, ruxolitinib was more expensive and more effective than BAT in 43.44% of the probabilistic iterations (Fig. 3).

### One-way sensitivity analysis 

**Fig. 4:**
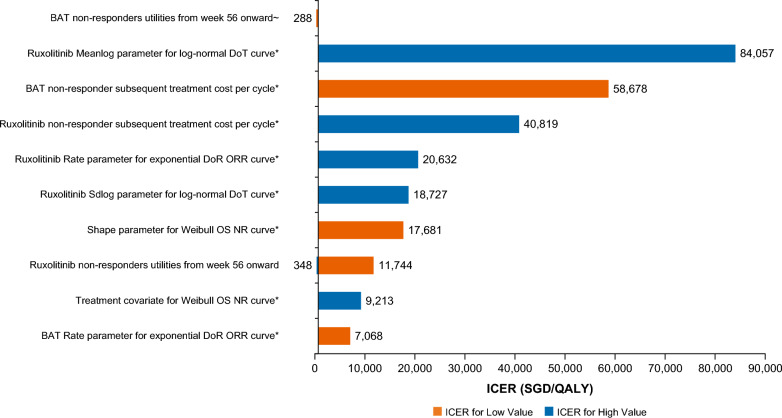
Tornado diagram for ICER based on one-way sensitivity analysis. *Dominant, ~ Dominated. BAT, best available therapy; DoR, duration of response; DoT, duration of treatment; ICER, incremental cost-effectiveness ratio; NR, non-responder; ORR, overall response rate/overall responder; OS, overall survival; QALY, quality-adjusted life year; SD, standard deviation; SGD, Singapore dollar

In the OWSA, a 20% increase in utilities for NR in the BAT arm from Week 56 onward led to BAT dominating (less expensive and more effective than) ruxolitinib (Fig. 4). A 20% increase in the meanlog parameter used to determine DoT for ruxolitinib also increased ICERs to SGD 84,057/QALY. All other variations did not lead to ICERs greater than SGD 75,000/QALY. Conversely, for eight of the ten greatest drivers of uncertainty, ruxolitinib dominated BAT when parameters were varied to favor ruxolitinib (Fig. 4 and Additional file 1: Table S7). Threshold values that caused a switch to dominant or dominated ICER values are presented in the Additional file 1: Table S7.

### Scenario analysis 

Multiple scenarios were considered to explore the sensitivity of cost-effectiveness findings to the underlying assumptions (Table 3). The appropriate curves were selected based on goodness of fit to the KM data and clinical plausibility of the predicted patient survival (alternate survival curves: Additional file 1: Fig. S1(d–i); AIC and BIC data on individual fit: Additional file 1: Table S2).

**Table 3:** Summary of additional deterministic scenario analyses

Scenario	Incremental LYs	Incremental QALYs	Incremental costs	ICER
Deterministic base case	0.86	0.80	SGD 540	SGD 677/QALY
Discount rate (0%)	1.63	1.41	SGD 4842	SGD 3446/QALY
Discount rate (5%)	0.59	0.58	SGD 32	SGD 55/QALY
Time horizon 30 years	0.69	0.68	− SGD 2888	Dominant
Time horizon 50 years	0.95	0.86	SGD 2726	SGD 3168/QALY
OS HR approach	0.72	0.70	SGD 4605	SGD 6593/QALY
OS combined fit alternate curve	0.64	0.63	− SGD 16,250	Dominant
DoT KM individual treatments	0.86	0.80	− SGD 31,454	Dominant
DoT KM by treatment arm	0.86	0.80	− SGD 33,187	Dominant
DoT KM then extrapolated by response	0.86	0.80	SGD 13,876	SGD 17,370/QALY
DoR alternative curve	0.86	0.70	SGD 49,472	SGD 70,758/QALY
Societal perspective	0.86	0.80	− SGD 27,960	Dominant
Age-adjusted utilities: regression equation	0.86	0.73	SGD 540	SGD 739/QALY
Age-adjusted utilities: Gen pop age category	0.86	0.81	SGD 540	SGD 669/QALY
Single comparator: ECP	0.70	0.68	− SGD 82,391	Dominant
Single comparator: MMF	0.72	0.70	SGD 63,741	SGD 91,199/QALY
Single comparator: MTX	1.02	0.91	SGD 76,198	SGD 83,448/QALY
BAT distribution based on informal physician survey	0.86	0.80	SGD 11,459	SGD 14,344/QALY
REACH3 BAT distribution	0.86	0.80	SGD 29,764	SGD 37,259/QALY
Include concomitant medicines	0.86	0.80	SGD 535	SGD 670/QALY
Include terminal care costs	0.86	0.80	SGD 666	SGD 833/QALY
Drug costs only	0.86	0.80	SGD 11,229	SGD 14,057/QALY

BAT, best available therapy; DoR, duration of response; DoT, duration of treatment; ECP, extracorporeal photopheresis; Gen pop, general population; HR, hazard ratio; ICER, incremental cost-effectiveness ratio; KM, Kaplan–Meier; LY, life year; MMF, mycophenolate mofetil; MTX, methotrexate; OS, overall survival; QALY, quality-adjusted life year; SGD, Singapore dollar

ICERs were observed to be greater than SGD 75,000/QALY when ruxolitinib was compared to single comparators mycophenolate mofetil (SGD 91,199/QALY) and methotrexate (SGD 83,448/QALY). ICERs remained lower than SGD 75,000/QALY for all the other scenarios explored. Ruxolitinib dominated BAT in the scenarios when: time horizon was set to 30 years; alternative clinically reasonable parametric survival curve was used for survival extrapolation; DoT was determined using Kaplan–Meier curves from the REACH3 trial either by individual treatment arm; a societal perspective was considered; or ruxolitinib was compared to ECP as a single comparator.

## Discussion

Allo-HSCT is a resource extensive procedure, costing approximately SGD 150,000 for a single patient [17]. To ensure the success of this procedure and maximize value gained, it is critical for patients to be able to access the most effective post-transplant supportive care. Despite significant progress in recent years, there remains an unmet need to improve long-term posttransplant outcomes of allo-HSCT recipients [20]. A key determinant of the long-term QoL of patients who undergo HSCT is the occurrence and severity of GVHD [20]. Patients with SR-cGVHD after allo-HSCT are significantly impacted, with the mean total cost after 2 years of the transplant increasing to more than double (United States dollar [USD] 532,673) compared with those without cGVHD (USD 252,909; *P* < 0.001) [21]. This may be attributed to the fact that patients who develop SR-cGVHD often require multiple additional therapies and long-term medical care (up to 75.3% of patients with SR-cGVHD need ≥ 4 lines of therapy) [21]. It is crucial that these patients gain access to the most effective options to optimize their treatment outcomes.

The approval of ruxolitinib offers a promising and novel treatment to meet the needs of patients with SR-cGVHD. In the REACH3 trial, ruxolitinib demonstrated a higher overall response than BAT at Week 24 (49.7% vs. 25.6%), higher best overall response (76.4% vs. 60.4%), longer DoR, and longer failure-free survival [22]. Patients treated with ruxolitinib had a greater reduction of symptoms compared with those in the control group, when measured using the GVHD-specific modified Lee Symptom Scale [22]. These outcomes have been correlated with better survival; however, longer-term follow-up data are essential to confirm long-term survival outcomes [22–24].

In this analysis, we synthesized results from the REACH3 trial into the Singaporean context, demonstrating that ruxolitinib is likely to be cost-effective compared with BAT for the treatment of SR-cGVHD over a 40-year time horizon. Gains in health were attributable to increased overall response to ruxolitinib compared with BAT, leading to 0.86 incremental LYs and 0.80 incremental QALYs. Ruxolitinib was found to be associated with additional costs of SGD 540 due to higher initial drug acquisition costs that were partially offset by lower subsequent treatment and healthcare resource utilization costs. In line with ACE guidance, this study did not account for (direct and indirect) nonmedical costs such as childcare, years of labor lost due to the disease, or its treatment [17]. This omission may have led to a more conservative estimate of the actual cost-effectiveness of ruxolitinib from a societal perspective.

Parameter uncertainty was explored using PSA, which found that ruxolitinib had a 78.52% probability of being cost-effective at a WTP threshold of SGD 75,000/QALY. We further aimed to investigate the robustness of model findings to several structural assumptions via OWSA and scenario analysis. Most of the parameter variations and scenarios explored were consistent with our base case, with ruxolitinib associated with ICERs lower than SGD 75,000/QALY and even dominating BAT in certain scenarios. OWSA revealed that our model was sensitive to variations in NR utility in the BAT arm, with a 20% increase in post-Day 56 BAT NR utilities, leading to BAT dominating ruxolitinib. The cost-effectiveness of ruxolitinib was sensitive to subsequent treatment costs. Variations to favoring BAT for DoT for the BAT arm (SGD 84,057/QALY), subsequent treatment costs for BAT (SGD 58,678/QALY), and subsequent treatment costs for RUX (SGD 40,819/QALY) were the next largest drivers of uncertainty in the OWSA.

The choice of comparator was also a significant factor that influenced the cost-effectiveness of ruxolitinib. Scenario analysis showed that ruxolitinib was associated with ICERs > SGD 75,000/QALY when compared against single comparators MMF (SGD 91,199/QALY) and MTX (SGD 83,448/QALY). These individual comparisons are highly uncertain as the REACH3 trial was not powered to investigate the differences in efficacy, AEs, or drug dosing between ruxolitinib and individual interventions. When compared with a treatment mix of interventions based on the composition of the BAT used in REACH3, ICERs associated with ruxolitinib (SGD 37,259/QALY) remained lower than SGD 75,000/QALY.

A previous cost-effectiveness study evaluating SR-cGVHD treatments in adult patients by Yalniz et al. compared the cost per response type (complete or partial) and cost per organ system-specific response [25], finding that ruxolitinib was associated with higher costs per overall response (USD 97,807) when compared with ECP (USD 67,400) and MTX (USD 453). There were several key differences in study designs that led to this discrepancy. In the previous study, researchers only considered costs of 6 months of drug acquisition. As patients with cGVHD require systemic immunosuppressive treatment for a median of 2–3 years, the analysis neglected the potential long-term cost offsets from treatment with ruxolitinib [25]. Indeed, as seen in our model, although ruxolitinib was associated with higher initial drug acquisition costs than BAT, these costs were offset over a longer time horizon. Furthermore, the study by Yalniz et al. did not consider differential mortality or QoL following response to treatment and did not holistically capture the impacts of achieving overall response in patients. Finally, the Yalniz et al. study was conducted prior to publication of the REACH3 findings, and comparison of efficacy between treatments relied on unanchored comparisons, introducing uncertainty about the validity of these comparisons. As such, we believe that our current analysis provides a more updated and comprehensive understanding of the cost-effectiveness of ruxolitinib for treatment of SR-cGVHD.

Despite our best efforts to present a comprehensive cost-effectiveness analysis, we acknowledge that our study faced several inherent limitations. Our model did not account for differential risks of mortality and complications attributable to heterogeneity of patient characteristics, disease subtypes or underlying diseases that necessitated initial treatment with allo-HSCT. Furthermore, AEs and complications captured in the model were not explicitly modeled through separate health states, but as a one-time average cost and disutility that was applied at the median time-to-event. Due to the limited sample size of REACH3 trial, a robust subgroup analysis was deemed to be unfeasible, however, these concerns are partially addressed as the survival curves collected during the REACH3 trial would have already captured the increased mortality associated with underlying disease or treatment-related complications. Future studies exploring the heterogeneity of responses to ruxolitinib would provide critical information that can aid in addressing these concerns in subsequent models.

In this model, we assumed that patients in the NR health-state would continue to be treated with BAT. Subsequent therapy prescribed after initial treatment with ruxolitinib or BAT is highly variable, and multiple options may be chosen depending on the patient. While we recognize that previous treatment would influence subsequent treatment choice, in this study we were unable to account for previous treatment when determining subsequent treatment due to limited availability of data on local treatment distributions. Uncertainty around subsequent treatment cost has been tested in our OWSA and at all values tested, ICERs remained lower than the WTP of 75,000 SGD/QALY. Future studies would benefit from employing a more comprehensive model capable of patient level analysis.

As long-term survival data from the REACH3 trial are immature, the accuracy of this model is dependent on the accuracy of the survival extrapolation. While we acknowledge the inherent uncertainty associated with such survival extrapolations, model findings were found to be robust in our uncertainty analysis. In the OWSA, although ICER values were sensitive to variations in parameters used to determine the survival extrapolation curve, none of the variations led to ICERs greater than SGD 75,000/QALY. Similarly, the scenario analysis, which explored alternative approaches to survival extrapolation, found that ICERs remained lower than SGD 75,000/QALY in all the tested scenarios.

While this study has found that ruxolitinib likely represents a cost-effective option for treatment of SR-cGVHD in Singapore, it does not provide information about the affordability of the intervention. To further substantiate our findings, a budget impact analysis could be conducted to provide information on the total costs of reimbursing this intervention to inform a healthcare payer’s reimbursement decision.

## Conclusions

SR-cGVHD is a major driver of mortality and morbidity following allo-HSCT. Supplementing the efficacy and safety findings from the REACH3 trial, we found that ruxolitinib is likely to be a cost-effective option for the treatment of Singaporean patients > 12 years of age who develop SR-cGVHD following allo-HSCT. Ruxolitinib shows promise in bridging the efficacy gap in the treatment landscape of this patient group, and we believe improving access to this drug will facilitate better outcomes for these patients.

## Supplementary Information

**Additional file 1. Table S1**: Choice of parametric models used for extrapolation in the base case and their respective formulas. **Table S2**: Parameters of model fit for parametric survival extrapolation. **Table S3**: Costs and resources used while treating patients with SR-cGVHD in Singapore (Singapore dollar). **Table S4**: Health state utility values. **Table S5**: Utility decrements and duration of event for disease complication and AE event. **Table S6**: List of scenarios and variables evaluated in the costeffectiveness model. **Table S7**: Variations in one way sensitivity analysis that cause a switch to dominant or dominated ICER. **Fig. S1**: Comparison of response outcomes.

## Data Availability

All data generated or analyzed during this study are included in this published article (and its supplementary information files).

## Abbreviations

ACE: Agency for care effectiveness
AE: Adverse event
AIC: Akaike’s information criterion
Allo: Allogeneic
ALT: Alanine aminotransferase
BAT: Best alternative therapy
BIC: Bayesian information criterion
cGVHD: Chronic graft-versus-host disease
CMV: Cytomegalovirus
DoR: Duration of response
DoT: Duration of treatment
ECP: Extracorporeal photopheresis
EQ-5D: EuroQoL five-dimensional
GVHD: Graft-versus-host disease
HR: Hazard ratio
HSCT: Hematopoietic stem cell transplantation
ICER: Incremental cost-effectiveness ratio
IPD: Individual patient-level data
JAK: Janus kinase
KM: Kaplan–Meier
LY: Life year
MMF: Mycophenolate mofetil
MTX: Methotrexate
NR: Non-responder
ORR: Overall responder
OS: Overall survival
OWSA: One-way sensitivity analysis
PSA: Probabilistic sensitivity analysis
QALY: Quality-adjusted life year
QoL: Quality of life
RUX: Ruxolitinib
SD: Standard deviation
SGD: Singapore dollar
SR: Steroid-refractory
USD: United States dollar
WTP: Willingness to pay
