# Supplementary material for: A cost-effectiveness analysis of ruxolitinib versus best alternative therapy for patients with steroid-refractory chronic graft-versus-host disease aged > 12 years in Singapore
Source: Cost Eff Resour Alloc. 2023 May 31;21:34. doi: 10.1186/s12962-023-00444-w (PMC10230805; doi:10.1186/s12962-023-00444-w)
Supplement: Supplementary file 1 — Additional file 1. Table S1: Choice of parametric models used for extrapolation in the base case and their respective formulas. Table S2: Parameters of model fit for parametric survival extrapolation. Table S3: Costs and resources used while treating patients with SR-cGVHD in Singapore (Singapore dollar). Table S4: Health state utility values. Table S5: Utility decrements and duration of event for disease complication and AE event. Table S6: List of scenarios and variables evaluated in the costeffectiveness model. Table S7: Variations in one way sensitivity analysis that cause a switch to dominant or dominated ICER. Fig. S1: Comparison of response outcomes. [file 12962_2023_444_MOESM1_ESM.docx]

SUPPLEMENTARY INFORMATION

**Figure S1** Comparison of response outcomes

Figure a-c show the survival models used in the base-case while figure d-i show all the survival curves overlayed with their respective Kaplan Meier Plots.

Abbreviations: BAT = best available therapy; KM = Kaplan-Meier; OS = overall survival.


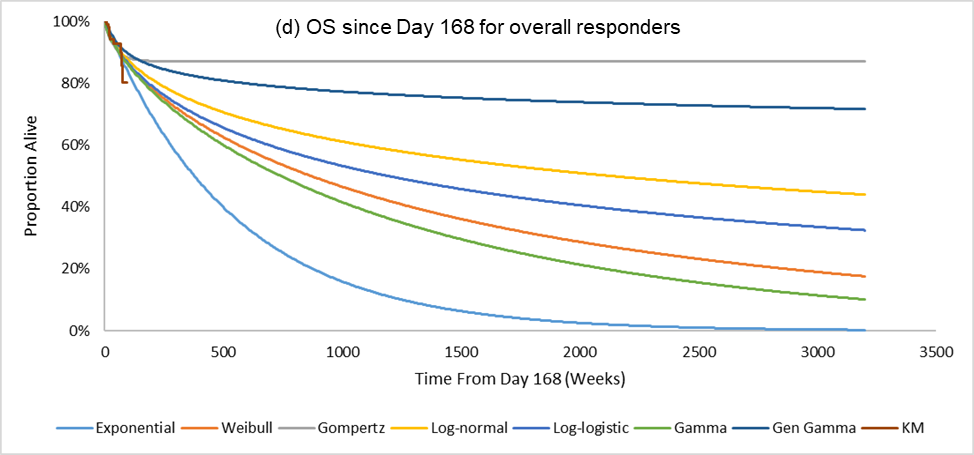


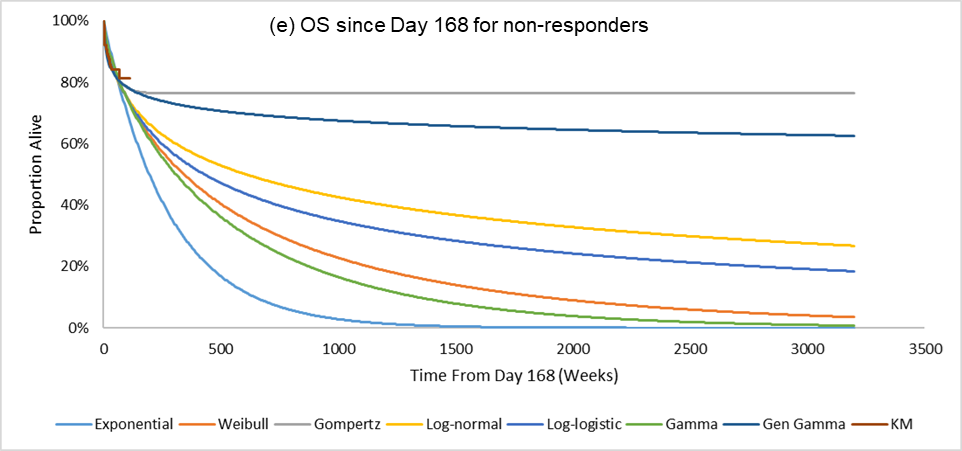


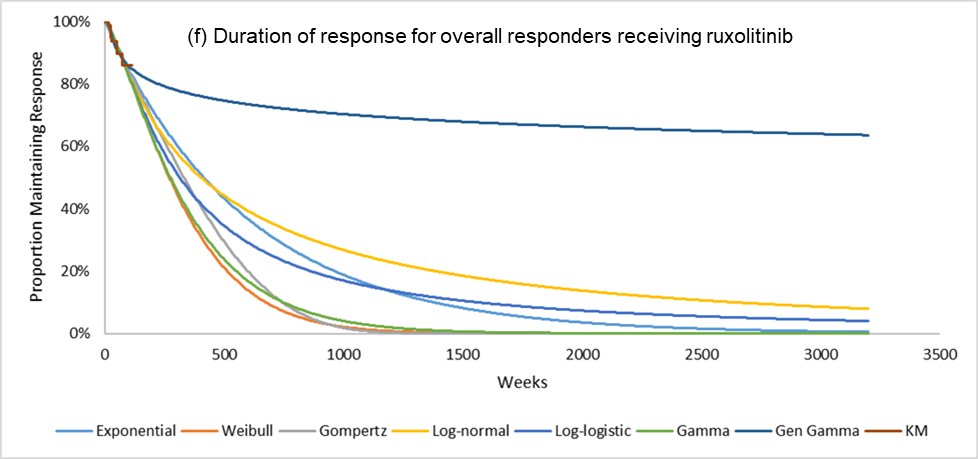


**
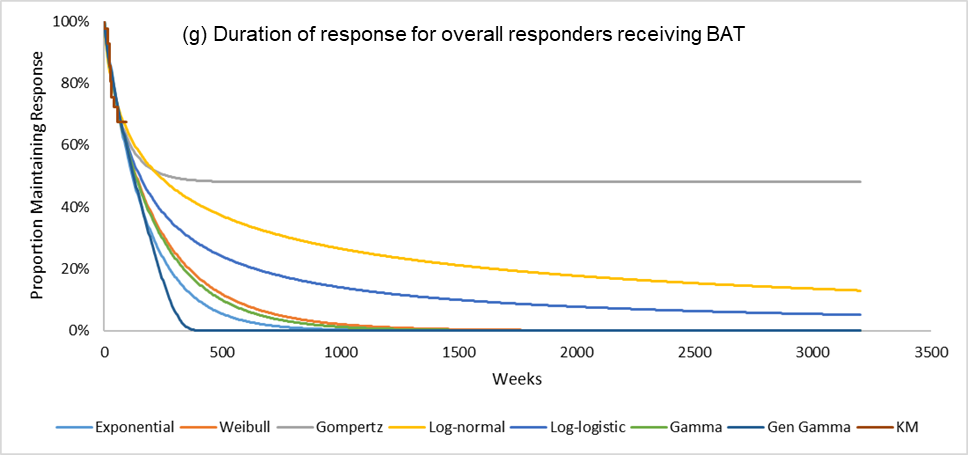
**


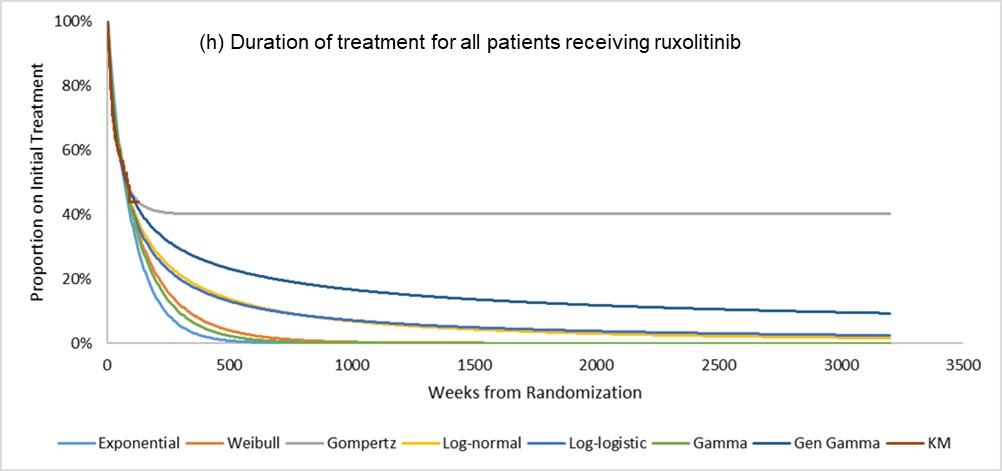


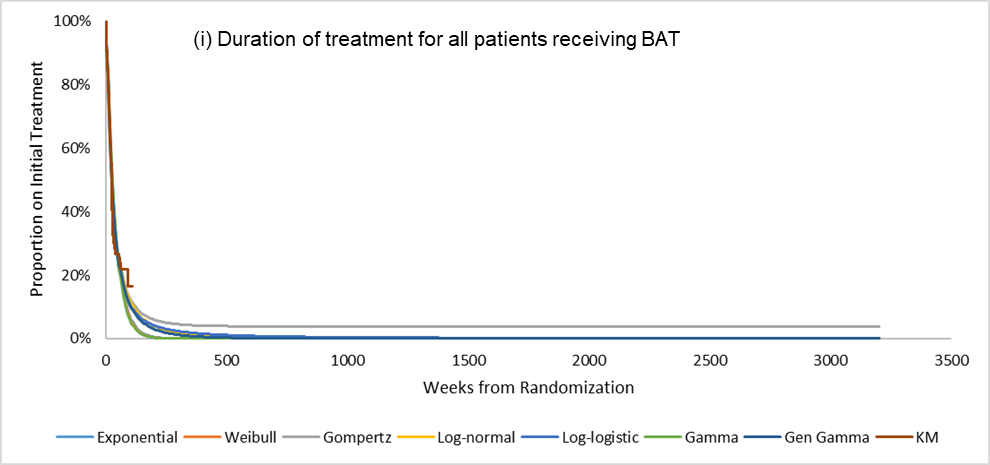


### **Table S1** Choice of parametric models used for extrapolation in the base case and their respective formulas

| **Model Parameter** | | **Survival curve used** | **Selection criteria** |
| --- | --- | --- | --- |
| ***Overall Survival*** | | | |
| OS curve type (combined fit^a^ for overall responders and non-responders) | | Weibull | Curve with best possible visual and statistical fit (AIC/BIC), and best clinical fit. |
| Duration of overall response curve type (ruxolitinib) | | Exponential |  |
| Duration of overall response curve type (BAT) | | Exponential |  |
| ***Drug Dosing and Duration of Treatment*** | | | |
| Duration of ruxolitinib treatment | | Log-normal | Curve with best possible visual and statistical fit (AIC/BIC), and best clinical fit. |
| Duration of BAT | | Log-logistic |  |
| **Formulas used for the Parametric survival functions** | | | |
|  | **Survival Function (Excel)** | | **Treatment Covariate Function (Excel)** |
| Exponential | $S\left( t \right)=\exp\left( -rate*t \right)$ | | 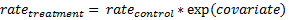 |
| Weibull | $S\left( t \right)=\exp\left( -scale{*t}^{shape} \right)$ | | 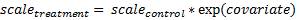 |
| Gompertz | $S\left( t \right)=\exp\left[ \left( \frac{\mathrm{rate}}{shape} \right)*\left( 1-exp(shape*t \right)) \right]$ | | 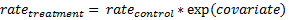 |
| Log-normal | $S\left( t \right)=1- NORMDIST \left( \frac{\ln\left( t \right)-meanlog}{sdlog} , 0, 1, TRUE \right)$ | | 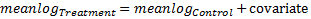 |
| Log-logistic | $S\left( t \right)=\frac{1}{1+ \exp\left( -\ln\left( scale \right)*shape \right)*t^{shape}}$ | | 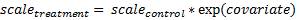 |
| Gamma | $S\left( t \right)= \Gamma(rate*t, shape)$ | | 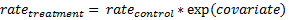 |
| Generalized gamma | $S\left( t \right)=1-GAMMADIST (\lambda*t^{\delta}, \gamma, 1, TRUE)$  Where:  $\lambda= \gamma*{exp(-mu)}^{\delta}$  $\gamma=\frac{1}{Q^{2}}$  $\delta=\frac{Q}{sigma}$ | | 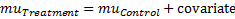 |

Abbreviations: BAT = best available therapy; OS = overall survival

^a^ To model OS in the base case using the combined fit approach, where data for both the overall responder and non-responder curves are used to find the optimal curve fit for both curves, a treatment covariate is calculated (i.e., relationship between the overall responder and non-responder curves)

### **Table S2** Parameters of model fit for parametric survival extrapolation.

| **Overall Survival Combined fit AIC and BIC** | | | | |
| --- | --- | --- | --- | --- |
| **Overall** | **AIC** | | | **BIC** |
| Exponential | 455.31 | | | 462.58 |
| Weibull | 455.31 | | | 462.58 |
| Gompertz | 450.50 | | | 461.39 |
| Log-normal | 447.22 | | | 458.11 |
| Log-logistic | 450.82 | | | 461.72 |
| Gamma | 452.24 | | | 463.14 |
| Generalised gamma | 441.43 | | | 455.96 |
| **Duration of Response individual fit AIC and BIC for Ruxolitinib** | | | | |
| **Ruxolitinib** | **AIC** | | | **BIC** |
| Exponential | 120.31 | | | 122.71 |
| Weibull | 121.64 | | | 126.45 |
| Gompertz | 122.29 | | | 127.11 |
| Log-normal | 120.51 | | | 125.32 |
| Log-logistic | 121.45 | | | 126.26 |
| Gamma | 121.51 | | | 126.32 |
| Generalised gamma | 119.91 | | | 127.13 |
| **Duration of Response individual fit AIC and BIC for BAT** | | | | |
| **BAT** | | **AIC** | **BIC** | |
| Exponential | | 149.64 | 158.10 | |
| Weibull | | 151.18 | 154.65 | |
| Gompertz | | 150.91 | 154.39 | |
| Log-normal | | 153.32 | 156.79 | |
| Log-logistic | | 151.10 | 154.58 | |
| Gamma | | 151.17 | 154.64 | |
| Generalised gamma | | 153.43 | 158.64 | |
| **Duration of Treatment individual fit AIC and BIC for Ruxolitinib** | | | | |
| **Ruxolitinib** | | **AIC** | **BIC** | |
| Exponential | | 835.53 | 838.63 | |
| Weibull | | 832.81 | 839.02 | |
| Gompertz | | 824.78 | 830.99 | |
| Log-normal | | 822.84 | 829.05 | |
| Log-logistic | | 827.45 | 833.66 | |
| Gamma | | 834.28 | 840.49 | |
| Generalised gamma | | 821.83 | 831.15 | |
| **Duration of Treatment individual fit AIC and BIC for BAT** | | | | |
| **BAT** | | **AIC** | **BIC** | |
| Exponential | | 992.62 | 995.67 | |
| Weibull | | 994.51 | 1000.61 | |
| Gompertz | | 990.86 | 996.96 | |
| Log-normal | | 985.61 | 991.71 | |
| Log-logistic | | 981.74 | 987.83 | |
| Gamma | | 993.72 | 999.81 | |
| Generalised gamma | | 987.22 | 996.37 | |

In the absence of direct clinical trial data comparing treatments, efficacy data for comparators were sourced from literature reviews. Health state utilities and disutilities used in the Crespo et al. study were also sourced from the literature, which were used in the de Waure et al. study as well. Both studies included only direct medical costs; however, the definitions of these costs varied between studies to include costs related to treatment, administration, and hospitalization.

### **Table S3** Costs and resources used while treating patients with SR-cGVHD in Singapore (SGD)

| **Drug acquisition unit costs** | | | | | |
| --- | --- | --- | --- | --- | --- |
| **Treatment** | **Formulation size** | | **Unit cost** | **Source of cost** | |
| Ruxolitinib | 5 mg | | SGD 41.3 | (Hypothetical cost) | |
| Rituximab | 100 mg | | SGD 82.25 | Provided by clinician collaborator | |
| ECP | 1 treatment | | SGD 2414 | Provided by clinician collaborator | |
| IMA | 400 mg | | SGD 15 | IMS data Aug 2021, Glivec® film-coated tablet 400 mg (data on file) | |
| MTX | 2.5 mg | | SGD 0.14 | Provided by clinician collaborator | |
| MMF | 360 mg | | SGD 4.18 | Provided by clinician collaborator | |
| Sirolimus | 1 mg | | SGD 11 | IMS data Aug 2021, Rapamune® tablet 1 mg (data on file) | |
| Ibrutinib | 140 mg | | SGD 103.70 | Provided by clinician collaborator | |
| **Treatment administration frequency and costs** | | | | | |
| **Treatment** | **Mode of administration** | | **Cost per administration** | **Source of cost** | |
| Rituximab | IV | | SGD 263.71 | Once per week for 4 weeks, followed by once per month | |
| ECP | NA | | SGD 120 | Provided by clinician collaborator | |
| **Non-responder weekly subsequent treatment costs** | | | | | |
| **Subsequent treatment cost type** | **After initial treatment with ruxolitinib** | | **After initial treatment with BAT** | Based on weighted average of treatments in BAT arm | |
| BAT drug acquisition | SGD 540.54 | | SGD 540.54 |  |  |
| BAT treatment administration | SGD 24.53 | | SGD 24.53 |  |  |
| **Annualized event rates and mean LOS by health state^2^** | | | | | |
| **Facility/visit type** | **Mean annualized event rate** | | | **Mean LOS** | |
|  | **Ruxolitinib** | | **BAT** | **Ruxolitinib** | **BAT** |
| ***Disease baseline*** | | | | | |
| BMT unit | 0.28 | | 0.63 | 15.86 | 15.84 |
| ER | 0.08 | | 0.28 | 1.00^a^ | 1.00^a^ |
| General ward | 0.83 | | 1.03 | 19.11 | 17.57 |
| ICU | 0.55 | | 0.60 | 9.31 | 18.15 |
| Specialist visit | 0.24 | | 0.15 | 1.00^b^ | 1.00^b^ |
| GP visit | 0.10 | | 0.02 | 1.00^b^ | 1.00^b^ |
| Urgent care visit | 0.06 | | 0.03 | 1.00^b^ | 1.00^b^ |
| ***Overall responders*** | | | | | |
| BMT unit | 0.05 | | 14.25 |  |  |
| ER | 0.08 | | 1.00^a^ |  |  |
| General ward | 0.43 | | 10.16 |  |  |
| ICU | 0.06 | | 17.86 |  |  |
| Specialist visit | 0.04 | | 1.00^b^ |  |  |
| GP visit | 0.03 | | 1.00^b^ |  |  |
| Urgent care visit | 0.08 | | 1.00^b^ |  |  |
| ***Non-responders*** | | | | | |
| BMT unit | 0.70 | | 16.03 |  |  |
| ER | 0.24 | | 1.00^a^ |  |  |
| General ward | 1.23 | | 23.84 |  |  |
| ICU | 0.90 | | 11.82 |  |  |
| Specialist visit | 0.29 | | 1.00^b^ |  |  |
| GP visit | 0.08 | | 1.00^b^ |  |  |
| Urgent care visit | 0.03 | | 1.00^b^ |  |  |
| **Unit costs for resource use** | | | | | |
| **Resource** | **Cost per day/visit** | | **Source/notes** | | |
| **BMT unit** | **SGD 436** | | ACE costing template (data on file)  *Follow general ward cost as Singapore does not have different costs for BMT wards; assume same costs for initial admission and readmission | | |
| ER | SGD 113.08 | | ACE costing template (data on file) | | |
| General ward | SGD 436 | | ACE costing template (data on file) | | |
| ICU | SGD 869.16 | | ACE costing template (data on file) | | |
| Specialist visit | SGD 101 | | ACE costing template (data on file) | | |
| GP visit | SGD 50 | | ACE costing template (data on file)  Woodlands Health urgent care fees, Link: <https://www.whc.sg/feelingunwell/urgent-care> | | |
| Urgent care visit | SGD 90 | |  |  |  |
| Terminal care costs | SGD 3210.90 | | Phua LC, Lee SC, Ng K, Abdul Aziz MI. Cost-effectiveness analysis of atezolizumab in advanced triple-negative breast cancer. BMC Health Serv Res 2020; 20:581. | | |
| **Disease complication and adverse event unit costs** | | | | | |
| **Additional event** | **Unit cost** | | **Source** | | |
| ***Disease complication*** | | | | | |
| cGVHD recurrence | SGD 202 | | Assumed temporary increase in monitoring represented by two additional specialist visits as suggested by clinician collaborator | | |
| ***Adverse event*** | | | | | |
| CMV infection | SGD 4,942 | | Average of IV Foscarnet for pre-emptive treatment of CMV infection: SGD390 per 6g (unit cost), given daily for 3 weeks + Oral valganciclovir (unit cost 450mg = SGD30.25): usual dose: 900mg BID x for average duration of 14 days | | |
| Thrombocytopenia | SGD 3,156.10 | | ACE costing template (data on file) | | |
| Anemia | SGD 1,486.00 | | ACE costing template (data on file) | | |
| Platelet count decreased | SGD 3,156.10 | | Assumed same as thrombocytopenia | | |
| Neutropenia | SGD 4,593.55 | | ACE costing template (data on file) | | |
| Pneumonia | SGD 1,2067.21 | | ACE costing template (data on file) | | |
| Leukopenia | SGD 4,593.55 | | Assumed same as neutropenia | | |
| ALT increased | SGD 2,117 | | https://www.ncbi.nlm.nih.gov/pmc/articles/PMC7941436/#cnr21308-supitem-0001 | | |
| **Societal perspective** | | | | | |
| **General Employment Parameters** | | | | | |
| **Parameters** | **Value** | | **Source** | | |
| Average Retirement Age in the General Population (Years) | 63 | | Retirement and Re-employment Act 1993 (2020 Revised edition). Singapore Statutes Online. Accessed: 10 May 2023. <https://sso.agc.gov.sg/Act/RRA1993> | | |
| Employment Rate in the General Population | 97.9% | | Labour, Employment, Wages and Productivity. Department of Statistics Singapore. Accessed: 10 May 2023. <https://www.singstat.gov.sg/find-data/search-by-theme/economy/labour-employment-wages-and-productivity/latest-data> | | |
| Average Salary **per Year** | SGD 42,648.00 | | Average and Median Monthly Household Income from Work Per Household Member Among Resident and Resident Employed Households. Department of Statistics Singapore. Accessed: 10 May 2023. <https://tablebuilder.singstat.gov.sg/table/CT/17812> | | |
| Average Hours Worked **per Week** | 46.2 | | Summary Table: Hours Worked. Ministry of Manpower. Accessed: 10 May 2023. <https://stats.mom.gov.sg/Pages/Hours-Worked-Summary-Table.aspx> | | |
| **Percentage of Work Hours Missed by Health State** | | | | | |
| **Health State** | **Ruxolitinib** | **BAT** | **Source** | | |
| Disease Baseline | 7.09% | 10.80% | Annualized rate of healthcare encounters by treatment arm from REACH 3 as percentage of year (i.e., 365.25 days) | | |
| Overall Responder | 1.75% | 1.75% | Annualized rate of healthcare encounters from REACH 3 for ORR as percentage of year (i.e., 365.25 days) | | |
| Non-Responder | 14.19% | 14.19% | Annualized rate of healthcare encounters from REACH 3 for NR as percentage of year (i.e., 365.25 days) | | |

^a^ LOS for ER facility was assumed to be 1 day because it was defined in the REACH3 dataset as ≤24 h.

^b^ The duration of each outpatient visit was assumed to be 1 day.

Abbreviations: ACE = Agency for Care Effectiveness; ALT = alanine aminotransferase; BAT = best available therapy; BMT = bone marrow transplant; cGVHD = chronic graft-versus-host disease; CMV = cytomegalovirus; ECP = extracorporeal photopheresis; ER = emergency room; GP = general practitioner; ICU = intensive care unit; IMA = imatinib; IMS = IQVIA IMS market research database ; IV = intravenous; LOS = length of stay; MMF = mycophenolate mofetil; MTX = methotrexate; NA = not available; SGD = Singapore dollar; SR-cGVHD = steroid-refractory chronic graft-versus-host disease.

### **Table S4** Health state utility values

| **Health state** | **Change from baseline** | **SE** | **Health state utility value** |
| --- | --- | --- | --- |
| Disease baseline | N/A | 0.017 | 0.66 |
| ***After assessment timepoint (Week 24)*** | | | |
| Overall responder | +0.06 | 0.023 | 0.72 |
| Non-responder | 0.00 | 0.027 | 0.66 |
| ***Week 56 onward*** | | | |
| Overall responder | +0.09 | 0.027 | 0.75 |
| Non-responder | +0.03 | 0.028 | 0.69 |

Abbreviations: N/A = not applicable; SE = standard error.

Source: Novartis (2020), REACH3 post hoc analyses of primary analysis (6-month data cut) individual patient data. Data on file.

### **Table S5** Utility decrements and duration of event for disease complication and AE

| **Additional event** | **Utility decrement** | **Data source/notes** | **Duration of event (days)** | **Source (OCC 2016/2017 MRD)** |
| --- | --- | --- | --- | --- |
| ***Disease complication*** | | | | |
| cGVHD recurrence | -0.040 | Crespo 2012 (difference between disease baseline and progressed) | 27.3 | T86001: GVHD (2017/2018 FOI) |
| ***AE*** | | | | |
| CMV infection | -0.218 | Stein 2018 (infection; not specific to CMV infection) | 11.9 | B250, B251, B258, B259, B271: CMV pneumonitis, hepatitis, other/unspecified, and mononucleosis (2017/2018 FOI) |
| Thrombocytopenia | -0.090 | Nafees 2008 | 6.0 | D695, D696: thrombocytopenia (secondary and unspecified) |
| Anemia | -0.090 | Beusterien 2010 | 4.7 | D649: anemia, unspecified |
| Platelet count decreased | -0.090 | Assumed same as thrombocytopenia | 6.0 | Assumed same as thrombocytopenia |
| Neutropenia | -0.090 | Nafees 2008 | 5.7 | D700: neutropenia |
| Pneumonia | -0.218 | Stein 2018 (infection; not specific to CMV infection) | 6.5 | J189: pneumonia, unspecified |
| Leukopenia | -0.090 | Nafees 2008 | 4.9 | D728: other specified disorder of white blood cells |
| ALT increased | 0.000 | Assumption (Wehler 2018) | 3.5 | R740: elevation of levels of transaminase and LDH |

Abbreviations: AE = adverse event; ALL = acute lymphoblastic leukemia; ALT = alanine aminotransferase; AML = acute myeloid leukemia; cGVHD = chronic graft-versus-host disease; CMV = cytomegalovirus; FOI = Freedom of Information; GVHD = graft-versus-host disease; LDH = lactic acid dehydrogenase; MDS = myelodysplastic syndrome; MRD = most responsible diagnosis; NHL = Non-Hodgkin lymphoma; OCC = Ontario Case Costing; SCT = stem cell transplant.

Sources:

Beusterien KM, Davies J, Leach M, Meiklejohn D, Grinspan JL, O'Toole A, et al. Population preference values for treatment outcomes in chronic lymphocytic leukaemia: a cross-sectional utility study. Health Qual Life Outcomes. 2010;8:50.

Crespo C, Pérez-Simón JA, Rodríguez JM, Sierra J, Brosa M. Development of a population-based cost-effectiveness model of chronic graft-versus-host disease in Spain. Clinical therapeutics. 2012 Aug 1;34(8):1774-87.

De Waure C, Capri S, Veneziano MA, Specchia ML, Cadeddu C, Di Nardo F, Ferriero AM, Gennari F, Hamilton C, Mancuso A, Quaranta G. Extracorporeal photopheresis for second-line treatment of chronic graft-versus-host diseases: results from a health technology assessment in Italy. Value in Health. 2015 Jun 1;18(4):457-66.

Jonesn’ CA, Fernandez L, Mesa OA, Weimersheimer P, Peters C. Burden of cost in chronic graft versus host disease following hematopoietic stem cell transplantation: predictions for the next decade. Value Health. 2015;18:A842.

Forsythe A, Brandt PS, Dolph M, Patel S, Rabe APJ, Tremblay G. Systematic review of health state utility values for acute myeloid leukemia. ClinicoEcon Outcomes Res. 2018;10:83–92.

Nafees B, Stafford M, Gavriel S, Bhalla S, Watkins J. Health state utilities for non small cell lung cancer. Health Qual Life Outcomes. 2008;6:84.

Stein EM, Yang M, Guerin A, Gao W, Galebach P, Xiang CQ, et al. Assessing utility values for treatment-related health states of acute myeloid leukemia in the United States. Health Qual Life Outcomes. 2018;16:193.

Wehler M, Storm M, Kowal S, Campbell C, Boscoe A. A health state utility model estimating the impact of ivosidenib on quality of life in patients with relapsed/refractory acute myeloid leukemia.<https://library.ehaweb.org/eha/2018/stockholm/215730/michael.storm.a.health.state.utility.model.estimating.the.impact.of.ivosidenib.html>. EHA Library. 2018.

### **Table S6** List of scenarios and variables evaluated in the cost-effectiveness model

| Scenario | Variable description | Default value | Alternate value for scenario analysis |
| --- | --- | --- | --- |
| Discount rate (0%) | Discount rate of costs  Discount rate of effects | 3.0% 3.0% | 0.0% 0.0% |
| Discount rate (5%) | Discount rate of costs  Discount rate of effects | 3.0% 3.0% | 5.0% 5.0% |
| Time horizon 30 years | Time horizon | 40 years | 30 years |
| Time horizon 50 years | Time horizon | 40 years | 50 years |
| OS HR approach | OS extrapolation methodology  BAT overall responders OS curve type  Ruxolitinib overall responders OS curve type  BAT non-responders OS curve type  Ruxolitinib non-responders OS curve type  OS HR: overall responders vs non-responders | Combined fit Weibull Weibull Weibull Weibull NA | HR Weibull Weibull Weibull Weibull 0.53 |
| OS combined fit alternate curve | OS extrapolation methodology  BAT overall responders OS curve type  Ruxolitinib overall responders OS curve type  BAT non-responders OS curve type  Ruxolitinib non-responders OS curve type | Combined fit Weibull Weibull Weibull Weibull | Combined fit Exponential Exponential Exponential Exponential |
| DoT KM individual treatments | Approach for modeling DoT | Extrapolated | KM for individual treatments |
| DoT KM by treatment arm | Approach for modeling DoT | Extrapolated | KM by treatment arm |
| DoT KM then extrapolated by response | Approach for modeling DoT BAT overall responders DoT curve type  Ruxolitinib overall responders DoT curve type  BAT non-responders DoT curve type Ruxolitinib non-responders DoT curve type | Extrapolated  NA NA NA NA | KM then extrapolated by response Log-normal Log-normal Log-normal Gamma |
| DoR alternative curve | BAT duration of overall response curve type  Ruxolitinib duration of overall response curve type | Exponential Exponential | Weibull Weibull |
| Societal perspective^a^ | Include indirect costs | No | Yes |
| Age-adjusted utilities: regression equation | Selection to adjust utilities by age | No | Regression equation by age |
| Age-adjusted utilities: general population age category | Selection to adjust utilities by age | No | By age category |
| Single comparator: ECP | Proportion of patients receiving RTX  Proportion of patients receiving ECP  Proportion of patients receiving IMA  Proportion of patients receiving MTX Proportion of patients receiving MMF Proportion of patients receiving EVE  Proportion of patients receiving SIR  Proportion of patients receiving IBR  Proportion of patients receiving INF  BAT overall response rate at Day 168  BAT complete response rate at Day 168 BAT partial response rate at Day 168  BAT no response rate at Day 168  Approach for modeling DoT | 5.0% 60.0% 0.0% 15.0% 15.0% 0.0% 0.0% 5.0% 0.0% 25.6% 3.0% 22.6% 68.3% Extrapolated | 0.0% 100.0% 0.0% 0.0% 0.0% 0.0% 0.0% 0.0% 0.0% 29.1% 1.8% 27.3% 64.8% KM for individual treatments |
| Single comparator: MMF | Proportion of patients receiving RTX  Proportion of patients receiving ECP  Proportion of patients receiving IMA  Proportion of patients receiving MTX Proportion of patients receiving MMF Proportion of patients receiving EVE  Proportion of patients receiving SIR  Proportion of patients receiving IBR  Proportion of patients receiving INF  BAT overall response rate at Day 168  BAT complete response rate at Day 168  BAT partial response rate at Day 168  BAT no response rate at Day 168  Approach for modeling DoT | 5.0% 60.0% 0.0% 15.0% 15.0% 0.0% 0.0% 5.0% 0.0% 25.6% 3.0% 22.6% 68.3% Extrapolated | 0.0% 0.0% 0.0% 0.0% 100.0% 0.0% 0.0% 0.0% 0.0% 28.6% 2.9% 25.7% 65.3% KM for individual Treatments |
| Single comparator: MTX | Proportion of patients receiving RTX  Proportion of patients receiving ECP Proportion of patients receiving IMA  Proportion of patients receiving MTX Proportion of patients receiving MMF Proportion of patients receiving EVE  Proportion of patients receiving SIR  Proportion of patients receiving IBR  Proportion of patients receiving INF  BAT overall response rate at Day 168  BAT complete response rate at Day 168 BAT partial response rate at Day 168  BAT no response rate at Day 168  Approach for modeling DoT | 5.0% 60.0% 0.0% 15.0% 15.0% 0.0% 0.0% 5.0% 0.0% 25.6% 3.0% 22.6% 68.3% Extrapolated | 0.0% 0.0% 0.0% 100.0% 0.0% 0.0% 0.0% 0.0% 0.0% 22.2% 7.4% 14.8% 71.7% KM for individual treatments |
| BAT composition based on informal physician survey | Proportion of patients receiving RTX  Proportion of patients receiving ECP Proportion of patients receiving IMA  Proportion of patients receiving MTX Proportion of patients receiving MMF Proportion of patients receiving EVE  Proportion of patients receiving SIR  Proportion of patients receiving IBR  Proportion of patients receiving INF | 5.0% 60.0% 0.0% 15.0% 15.0% 0.0% 0.0% 5.0% 0.0% | 40.0% 0.0% 0.0% 0.0% 40.0% 0.0% 0.0% 20.0% 0.0% |
| BAT composition based on REACH3 distribution | Proportion of patients receiving RTX  Proportion of patients receiving ECP Proportion of patients receiving IMA  Proportion of patients receiving MTX Proportion of patients receiving MMF Proportion of patients receiving EVE  Proportion of patients receiving SIR  Proportion of patients receiving IBR  Proportion of patients receiving INF | 5.0% 60.0% 0.0% 15.0% 15.0% 0.0% 0.0% 5.0% 0.0% | 9.8% 33.9% 9.2% 6.7% 25.4% 0.0% 9.2% 5.7% 0.0% |
| Include concomitant medicines | Include concomitant medication costs | Yes | No |
| Include terminal care costs | Include terminal care costs | Yes | No |
| Drug costs only | Include administration costs  Include AE costs  Include concomitant medication costs  Include indirect costs  Include resource use costs  Include terminal care costs | Yes Yes Yes No Yes Yes | No  No  No  No  No  No |

Abbreviations: AE = adverse event; BAT = best available therapy; DoR = duration of response; DoT = duration of treatment; ECP = extracorporeal photopheresis; EVE = everolimus; HR = hazard ratio; IBR = ibrutinib; IMA = imatinib; INF = infliximab; KM = Kaplan-Meier; MMF = mycophenolate mofetil; MTX = methotrexate; NA = not available; OS = overall survival; RTX = rituximab; SIR = sirolimus.

^a^ Societal perspective included lost earnings from early death and lost productivity from work missed due to illness.

### **Table S7** Variations in one way sensitivity analysis that cause a switch to dominant or dominated ICER.

| **Variable** | **Low Value** | **High Value** | **Dominated/ Dominant ICER Values** | **Low/High value causing dominated/ dominant ICER Values** |
| --- | --- | --- | --- | --- |
| BAT non-responders utilities from week 56 onward~ | 0.552 | 0.828 | Dominated | Dominated ICER is corresponding to high value of 0.828 |
| Ruxolitinib Meanlog parameter for log-normal DoT curve* | 3.444 | 5.166 | Dominant | Dominant ICER is corresponding to low value of 3.444 |
| BAT non-responder subsequent treatment cost per cycle* | SGD 1,729.74 | SGD 2,594.61 | Dominant | Dominant ICER is corresponding to high value of SGD 2,594.61 |
| Ruxolitinib non-responder subsequent treatment cost per cycle* | SGD 1,729.74 | SGD 2,594.61 | Dominant | Dominant ICER is corresponding to low value of SGD 1,729.74 |
| Ruxolitinib Rate parameter for exponential DoR ORR curve* | 0.001 | 0.002 | Dominant | Dominant ICER is corresponding to low value of 0.001 |
| Ruxolitinib Sdlog parameter for log-normal DoT curve* | 1.399 | 2.099 | Dominant | Dominant ICER is corresponding to low value of 1.399 |
| Shape parameter for Weibull OS NR curve* | 0.564 | 0.847 | Dominant | Dominant ICER is corresponding to high value of 0.847 |
| Treatment covariate for Weibull OS NR curve* | -0.527 | -0.79 | Dominant | Dominant ICER is corresponding to low value of -0.527 |
| BAT Rate parameter for exponential DoR ORR curve* | 0.005 | 0.007 | Dominant | Dominant ICER is corresponding to high value of 0.007 |
| Scale parameter for Weibull OS NR curve* | 0.009 | 0.014 | Dominant | Dominant ICER is corresponding to high value of 0.014 |
